# Behandlung des CD30‐positiven kutanen T‐Zell‐Lymphoms mit Brentuximab vedotin und Strahlentherapie – retrospektive multizentrische Analyse

**DOI:** 10.1111/ddg.15897_g

**Published:** 2026-04-08

**Authors:** Patrick Schummer, Caroline Glatzel, Philipp Schrüfer, Ingulf Lawrenz, Gabor Dobos, Ulrike Wehkamp, Svea Hüning, René Stranzenbach, Jan P. Nicolay, Matthias Goebeler, Marion Wobser

**Affiliations:** ^1^ Klinik für Dermatologie Venerologie und Allergologie Universitätsklinikum Würzburg Würzburg Deutschland; ^2^ Skinmed AG Lenzburg Schweiz; ^3^ Klinik und Poliklinik für Strahlentherapie und Radioonkologie Universitätsklinikum Würzburg Würzburg Deutschland; ^4^ Klinik für Dermatologie Venerologie und Allergologie Charité – Universitätsmedizin Berlin Berlin Deutschland; ^5^ Klinik für Dermatologie Venerologie und Allergologie Universitätsklinikum Schleswig‐Holstein Campus Kiel Kiel Deutschland; ^6^ Medical School Hamburg Hamburg Deutschland; ^7^ Hautklinik Klinikum Dortmund gGmbH Dortmund Deutschland; ^8^ Hautarztpraxis Schwelm Schwelm Deutschland; ^9^ Dermatologie‐Gevelsberg Gevelsberg Deutschland; ^10^ Klinik für Dermatologie Venerologie und Allergologie Universitätsklinikum Mannheim Mannheim Deutschland

**Keywords:** Brentuximab vedotin, kutanes T‐Zell‐Lymphom, Strahlentherapie, Brentuximab vedotin, cutaneous T‐cell lymphoma, radiotherapy

## Abstract

**Hintergrund und Zielsetzung:**

Während Brentuximab vedotin (BV) oder Strahlentherapie (RTx) gängige Behandlungsoptionen für CD30‐positive kutane T‐Zell‐Lymphome (CTCL) sind, wurde über die Wirksamkeit und Verträglichkeit ihrer gleichzeitigen oder aufeinanderfolgenden Anwendung nur selten berichtet. In dieser retrospektiven Analyse haben wir daher die Kombination von BV und RTx bei CD30‐positiven CTCL‐Patienten untersucht.

**Patienten und Methodik:**

Wir schlossen 14 CD30‐positive CTCL‐Patienten aus sechs deutschen Tumorzentren ein, die mit BV behandelt wurden; eine RTx wurde innerhalb eines Zeitraums von 3 Monaten vor und bis zu 3 Monaten nach der BV‐Behandlung durchgeführt. Die RTx wurde hauptsächlich als niedrig dosiertes Schema angewendet.

**Ergebnisse:**

Unerwünschte Ereignisse jeden Schweregrades traten bei 71% der Patienten auf, darunter häufige Nebenwirkungen wie periphere Neuropathie, Neutropenie und Radiodermatitis. Dreizehn Patienten erreichten eine vollständige oder partielle Remission als bestes Gesamtansprechen, jedoch zeigten 50% aller Patienten ein Fortschreiten der Erkrankung. Bei einer medianen Nachbeobachtungszeit von 14,4 Monaten betrug das mediane progressionsfreie Überleben 12,0 Monate, mit einer 1‐Jahres‐Rate von 34,0%.

**Schlussfolgerungen:**

Die simultane oder sequenzielle Therapie mit BV und RTx war machbar und wurde gut vertragen. Künftige randomisierte Untersuchungen sind erforderlich, um die Vorteile dieses kombinierten Behandlungskonzeptes sowie die angemessene Dosierung von BV und RTx prospektiv zu ermitteln.

## EINLEITUNG

Primäre kutane T‐Zell‐Lymphome (CTCL), deren häufigster Subtyp die Mycosis fungoides (MF) ist, stellen eine Untergruppe der extranodalen Non‐Hodgkin‐Lymphome (NHL) dar.^1^ Gängige Behandlungsmöglichkeiten des CTCL richten sich nach dem Tumorstadium und umfassen sowohl läsionsgerichtete Therapien (zum Beispiel topische Steroide, Chlormethin‐Gel, Bestrahlung, UV‐Lichttherapie) als auch systemische Behandlungen wie Methotrexat (MTX), Bexaroten oder Mogamulizumab.^2^ Brentuximab vedotin (BV), bestehend aus einem monoklonalen Anti‐CD30‐Antikörper und Monomethyl‐Auristatin E (MMAE), bietet eine wirksame Behandlung für das CD30‐positive CTCL.^3^ In der Phase‐III‐Studie ALCANZA (NCT01578499) wurde BV mit MTX oder Bexaroten nach Wahl des Arztes (physician's choice [PC]) verglichen.^3^ Die Ergebnisse zeigten ein medianes progressionsfreies Überleben (PFS) von 16,7 Monaten (95%‐Konfidenzintervall [KI] 15,4–21,6) für BV gegenüber 3,5 Monaten (95%‐KI 2,4–4,6) für PC. Darüber hinaus betrug die mediane Zeit bis zur nächsten Behandlung 13,4 Monate (95%‐KI 11,4–15,3) für BV im Vergleich zu 5,6 Monaten (95%‐KI 3,4–7,2) für PC.^4^ Auf der Grundlage dieser Ergebnisse hat die *Europäische Arzneimittelagentur* (EMA) BV zur Behandlung des CD30‐positiven CTCL als Zweitlinientherapie nach einer vorausgehender systemischen Behandlung zugelassen

Da CTCL äußert strahlenempfindlich sind, ist die Strahlentherapie (RTx) eine weitere wichtige therapeutische Option. Je nach Krankheitsstadium kann die RTx entweder kurativ oder in palliativer Intention eingesetzt werden. Lokale Bestrahlung und Ganzhautelektronenbestrahlung (total skin electron beam therapy; TSEBT) sind die am häufigsten eingesetzten Techniken.^5^, ^6^, ^7^ In letzter Zeit hat sich die niedrig dosierte RTx als bevorzugtes Behandlungsschema durchgesetzt.^8^ Georgakopoulos et al. behandelten 14 Patienten mit niedrig dosierter TSEBT und konnten dabei eine Gesamtansprechrate (overall response rate; ORR) von 92,3% (13/14) feststellen, wobei drei Patienten (21,4%) eine komplette Remission (complete remission; CR) erreichten. Es wurden keine schwerwiegenden Nebenwirkungen berichtet.^9^ Jüngste klinische Studien untersuchen eine gleichzeitige und/oder sequenzielle RTx mit systemischen Behandlungen wie BV. In einer klinischen Phase‐I‐Studie (NCT2822586) wurde BV in gleichzeitiger oder sequenzieller Kombination mit niedrig dosierter TSEBT bei fünf Patienten mit CD30‐positivem MF im Stadium IB bis IVA und Sézary‐Syndrom (SS) untersucht. Aufgrund der geringen Rekrutierungsrate nach Zulassung von BV für das CD30‐positive CTCL wurde die Studie abgebrochen. Die im Jahr 2020 auf der Tagung der *American Society of Clinical Oncology* (ASCO) vorgestellten Daten zeigten weder signifikant erhöhte Hauttoxizitäten noch ein verlängertes Therapieansprechen im Vergleich zu einer BV‐Monotherapie.^10^ Eine weitere, sich noch in der Rekrutierung befindliche Phase‐II‐Studie untersucht die gleichzeitige Anwendung einer ultraniedrig dosierten TSEBT und Brentuximab vedotin bei Patienten mit Mycosis fungoides und SS im Stadium I–IV (NCT05357794). Zum Zeitpunkt der Datenerhebung lagen noch keine Ergebnisse vor.

Bislang berichteten nur wenige Fallserien über BV in Kombination mit RTx, zumeist von Patienten mit nodalem (Non‐)Hodgkin‐Lymphom und einer Einleitung von BV/RTx nach einer Polychemotherapie.^11^, ^12^, ^13^, ^14^ Wir präsentieren hier eine multizentrische Kohorte von 14 CD30‐positiven CTCL‐Patienten aus sechs verschiedenen Tumorzentren, die im Rahmen des deutschen Netzwerks für kutane Lymphome BV mit einer sequenziellen oder simultanen RTx erhielten. Unser Hauptziel war es, die Verträglichkeit dieser sequenziellen/simultanen Therapie zu untersuchen, um festzustellen, ob diese Behandlungskombination für weitere, systematischere Untersuchungen in Frage kommt. Darüber hinaus wurden die Ansprechraten und die Dauer des Ansprechens in unserer Patientenkohorte in einem Real‐World‐Setting analysiert.

## PATIENTEN UND METHODIK

### Patienten

In diese retrospektive multizentrische Analyse wurden 14 CD30‐positive CTCL‐Patienten aus sechs verschiedenen Tumorzentren des deutschen Netzwerks für kutane Lymphome eingeschlossen. Alle erhielten BV im Rahmen der Zulassung sowie eine RTx. Letztere wurde entweder zwischen den BV‐Gaben (simultan) oder innerhalb von 3 Monaten vor Beginn beziehungsweise nach Absetzen von BV (sequenziell) eingeleitet. Zu den strahlentherapeutischen Modalitäten gehörten lokalisierte RTx oder TSEBT, ohne Beschränkung auf bestimmte Strahlendosen. Demografische und klinisch‐pathologische Merkmale, nachfolgende Therapien und unerwünschte Ereignisse (adverse events; AE) wurden aus elektronischen Krankenakten in einem Real‐World‐Setting extrahiert. Die Studie wurde gemäß den ethischen Richtlinien und der Deklaration von Helsinki durchgeführt und von der Ethikkommission genehmigt.

### Methodik

Die Stadieneinteilung des CTCL basierte auf den im Jahr 2007 von der *European Organisation for Research and Treatment of Cancer* (EORTC) und der *International Society for Cutaneous Lymphomas* (ISCL) überarbeiteten Stadieneinteilungen für Mycosis fungoides und Sézary‐Syndrom.^15^ Therapiebedingte Nebenwirkungen wurden gemäß *den Common Terminology Criteria for Adverse Events* (CTCAE) Version 5.0 des *National Cancer Institute* eingestuft. Das Therapieansprechen auf das kombinierte Behandlungsschema wurde definiert als: *(1)* komplettes Ansprechen (CR) bei vollständigem Rückgang aller klinischen CTCL‐Manifestationen, *(2)* partielles Ansprechen (partial response; PR) als Rückbildung aller messbaren Erkrankungsherde ohne Anzeichen einer voranschreitenden Erkrankung (progressive disease; PD), *(3)* stabile Erkrankung (stable disease; SD), wenn weder CR/PR noch PD in allen von der Krankheit betroffenen Läsionen erreicht wurden, und (*4*) PD bei Tumorprogression in einem Organsystem (zum Beispiel neue Hauttumorläsionen oder befallene Lymphknoten). Das globale Ansprechen wurde bei Absetzen von BV oder bei letzter Nachbeobachtung (je nachdem, was zuerst eintrat) evaluiert. Das beste Gesamtansprechen (best overall response; BOR) wurde als CR oder PR nach Beginn von BV definiert. Das PFS wurde von der Einleitung von BV bis zum Fortschreiten der Erkrankung nach der Kaplan‐Meier‐Methode berechnet. Die Zensierung erfolgte bei Tod oder letzter Nachbeobachtung. Der Median der Nachbeobachtungszeit wurde mit dem invertierten Kaplan‐Meier‐Ansatz berechnet (Datenstichtag 30. September 2021). Alle Analysen wurden mit R Version 4.2.2 durchgeführt (R‐Pakete survival, survminer, ggplot2, ranger, prodlim).

## ERGEBNISSE

### Basischarakteristika

Dreizehn Patienten hatten eine MF, davon waren acht Patienten im Erkrankungsstadium IIB und fünf im Stadium IV. Ein Patient litt an einem primären kutanen follikulären T‐Helferzell‐Lymphom (PCTL‐Tfh) ohne verfügbares Staging. Das mittlere Alter aller Patienten lag bei 57,5 Jahren (Bereich 45–85). Alle Patienten erhielten mindestens eine vorausgehende Systemtherapie, 50% der Patienten erhielten zwei oder mehr vorherige Systemtherapien. Zu diesen Therapien gehörten Bexaroten (12/14), MTX (6/14), systemische PUVA (Psoralene plus UVA‐Therapie) (3/14), Fumarsäureester (1/14), Gemcitabin (1/14) und Interferon alfa‐2a/b (1/14). Ein Patient (ID 8) erhielt im Vorfeld eine Therapie mit BV (der Abstand zwischen den unabhängigen BV‐Behandlungen betrug mehr als 6 Monate) und erreichte hierbei eine anfängliche CR. Die Basisdaten sind in Tabelle 1 aufgeführt.

**TABELLE 1:** Basischarakteristika.

	Patienten n/n (%)
***Geschlecht***	
Weiblich	3/14 (21)
Männlich	11/14 (79)
Subtyp des CTCL	
MF	13/14 (93)
PCTL‐Tfh	1/14 (7)
***Stadium (EORTC/ISCL 2007)***	
IIB	8/14 (57)
IVA1	2/14 (14)
IVA2	1/14 (7)
IVB	2/14 (14)
NA^*^	1/14 (7)
***Systemtherapie vor Beginn BV***	14/14 (100)
≥ 2 vorausgehende Systemtherapien	7/14 (50)
Brentuximab vedotin	1/14 (7)
Methotrexat	6/14 (43)
Bexaroten	12/14 (86)
Fumarsäureester	1/14 (7)
Systemische PUVA	3/14 (21)
Gemcitabin	1/14 (7)
Interferon alfa‐2a/b	1/14 (7)
***Medianes Alter bei Einleitung BV (Jahre) (Bereich)***	57,5 (45–85)

Prozentwerte ergeben möglicherweise nicht genau 100%, da gerundet wurde.

* Tumorstaging für PCTL‐Tfh nicht verfügbar.

*Abk*.: BV, Brentuximab vedotin; CTCL, kutanes T‐Zell‐Lymphom; EORTC, European Organisation for Research and Treatment of Cancer; ISCL, International Society of Cutaneous Lymphoma; MF, Mycosis fungoides; NA, nicht verfügbar; PCTL‐Tfh, primär kutanes follikulärer T‐Helferzell‐Lymphom; PUVA, Psoralen und ultraviolettes Licht A

### Therapeutischer Verlauf

Alle Patienten erhielten mindestens zwei Gaben von Brentuximab vedotin (Median 9,5 Zyklen [Bereich 2–16]). Die Dosierung von BV betrug, gemäß der Zulassung, mehrheitlich 1,8 mg/kg Körpergewicht (12/14), wurde aber bei der Hälfte der Patienten entsprechend den Behandlungsrichtlinien geändert (Tabelle 2). Brentuximab vedotin wurde bei allen Patienten mindestens einmal alle 3 Wochen (q3w) verabreicht, wobei eine Änderung des Intervalls (zum Beispiel einmal alle 6 Wochen) bei 21% (3/14) erforderlich war. Der Hauptgrund für eine zusätzliche RTx waren bei 79% (11/14) der Patienten neue Tumorläsionen. Weitere Gründe waren BV‐resistente Tumorläsionen, ein ulzerierter Tumor und ein allgemeines Fortschreiten der Krankheit. Bei den meisten Patienten (11/14) wurde eine lokale RTx durchgeführt, wobei drei Patienten eine TSEBT erhielten. Die RTx wurde bei 29% (4/14) vor der ersten Gabe von BV und bei 50% (7/14) nach Absetzen von BV eingeleitet. Die medianen Zeitabstände betrugen 11,5 Tage (Bereich 3–62) beziehungsweise 31 Tage (Bereich 18–67). Fünf Patienten erhielten eine RTx simultan zu BV, wie oben definiert. Die medianen Zeitunterschiede zwischen dem Beginn von BV und dem Beginn der RTx beziehungsweise dem Ende der RTx und dem Ende von BV betrugen 148,5 Tage (Bereich 13–249) beziehungsweise 82,5 Tage (Bereich 3–216). Es lagen keine Informationen dazu vor, ob die Behandlungen am selben Tag durchgeführt wurden. Zwei der Patienten (ID 6 und 8) erhielten jeweils zwei unabhängige RTx‐Zyklen in zeitlichem Zusammenhang mit BV: *(1)* ID 6 erhielt eine RTx simultan und 26 Tage nach dem letzten BV‐Zyklus, *(2)* ID 8 erhielt eine RTx 62 Tage vor und 31 Tage nach der BV‐Behandlung.

**TABELLE 2:** Therapeutischer Verlauf.

	Patienten n/n (%)
***Mediane Anzahl an BV‐Gaben (Anzahl) (Bereich)***	9,5 (2–16)
***Dosierung von BV***	
1,8 mg/kg KG	12/14 (86)
Dosismodifikation während BV	7/14 (50)
***Therapieintervalle von BV***	
Q3W	14/14 (100)
Intervallanpassung während BV	3/14 (21)
***Art der Strahlentherapie***	
Lokale Strahlentherapie	11/14 (79)
TSEBT	3/14 (21)
Gesamtstrahlendosis ≤ 12 Gy	8/14 (57)
***Beginn der Strahlentherapie*** ^*^	
≤ 3 Monate vor Einleitung BV	4/14 (29)
Mediane Zeit Start RTx und Start BV (Tage) (Bereich)	11.5 (3–62)
≤ 3 Monate nach Beendigung BV	7/14 (50)
Mediane Zeit Stopp BV und Start RTx (Tage) (Bereich)	31 (18–67)
Simultan zu BV	5/14 (36)
Mediane Zeit Start BV und Start RTx (Tage) (Bereich)	148.5 (13–249)
Mediane Zeit Stopp RTx und Stopp BV (Tage) (Bereich)	82.5 (3–216)
***Bestes Gesamtansprechen***	
CR	2/14 (14)
PR	11/14 (79)
PD	1/14 (7)
***Globales Ansprechen innerhalb des individuellen Beobachtungszeitraumes***	
CR	2/14 (14)
PR	4/14 (29)
SD	1/14 (7)
PD	7/14 (50)
***Anschlusstherapie*** ^**^	9/14 (64)
Mediane Nachbeobachtungszeit (Monate) (IQR)	14,4 (8,0–24,0)
Progressionsfreies Überleben	
Medianes progressionsfreies Überleben (Monate) (95%‐KI)	12,0 (7,3–NA)
1‐Jahres‐Rate (%) (95%‐KI)	34,0 (12,9–90,1)

Prozentwerte ergeben möglicherweise nicht genau 100%, da gerundet wurde.

* Ein Patient erhielt eine sequenzielle und eine simultane Bestrahlung, ein anderer Patient erhielt eine Strahlentherapie vor und nach der Therapie mit Brentuximab vedotin.

** Anschlusstherapien beinhalteten Mogamulizumab, Gemcitabin, Methotrexat, Psoralen plus ultraviolettes Licht A (PUVA), Bexaroten, pegyliertes liposomales Doxorubicin, Ganzhautelektronenbestrahlung and Resminostat.

*Abk*.: BV, Brentuximab vedotin; CR, Komplettremission; IQR, Interquartilbereich; KG, Körpergewicht; KI, Konfidenzintervall; NA, nicht verfügbar; PD, voranschreitende Erkrankung; PR, partielle Remission; Q3W, alle drei Wochen; RTx, Strahlentherapie; SD, stabile Erkrankung; TSEBT, Ganzhautelektronenbestrahlung

Die Bestrahlungsprotokolle waren heterogen, mit Einzeldosen von 8 Gy bis 30 Gy. Die meisten Patienten erhielten niedrig dosierte Schemata (Gesamtstrahlendosis ≤ 12 Gy). Hochdosistherapien (30 Gy) erhielten fünf Patienten, davon drei mit jeweils 10 x 3 Gy. Sechs Patienten erhielten eine Gesamtstrahlendosis von 8 Gy (2 x 4 Gy). Zusätzlich wurden fünf Patienten mit 2‐Gy‐Einzeldosen behandelt, die Gesamtdosen betrugen hierbei zwischen 12 Gy und 30 Gy. Dabei erhielten zwei Patienten eine Gesamtdosis von 24 Gy und 22 Gy, wobei letzterer die Bestrahlung aufgrund von AE vorzeitig abbrechen musste. Ein Patient erhielt eine Gesamtstrahlendosis von 30,6 Gy, wobei die einzelnen Fraktionen nicht näher spezifiziert wurden. Alle Patienten mit lokalisierter RTx erreichten eine CR in den bestrahlten Läsionen. Bei der TSEBT wurde eine Gesamtstrahlendosis von 12 Gy verabreicht, wobei unterschiedliche Einzeldosen von 1,5 Gy, 2 Gy und 4 Gy verwendet wurden. Bei zwei Patienten war ein zusätzlicher Boost für einzelne oder separate Tumorläsionen erforderlich. Das Ansprechen nach TSEBT in den bestrahlten Läsionen betrug PR für zwei Patienten und CR für einen Patienten.

Bezogen auf die Kombination von BV und RTx zeigten 13 Patienten zunächst ein Therapieansprechen, wobei 14% (2/14) eine CR als BOR aufwiesen. Bei 50% (7/14) kam es jedoch unter Therapie mit BV oder nach Absetzen von BV zu einem Fortschreiten der Krankheit. Diese Patienten erhielten eine RTx entweder nach BV (2/7), zwischen den BV‐Gaben (2/7) oder vor BV (1/7). Zwei weitere Patienten erhielten eine RTx *(1)* zwischen den BV‐Gaben und nach Absetzen von BV (ID 6) oder *(2)* sowohl vor als auch nach BV (ID 8). Ein globales Ansprechen in Form von CR oder PR wurde bei 43% (6/14) beobachtet. Hiervon erhielten jeweils zwei Patienten eine RTx vor, während oder nach der BV‐Behandlung. Zwei Patienten mit CR als BOR erhielten die RTx vor der BV‐Einleitung und zeigten eine anhaltende CR während der Nachbeobachtungszeit.

**ABBILDUNG 1:**
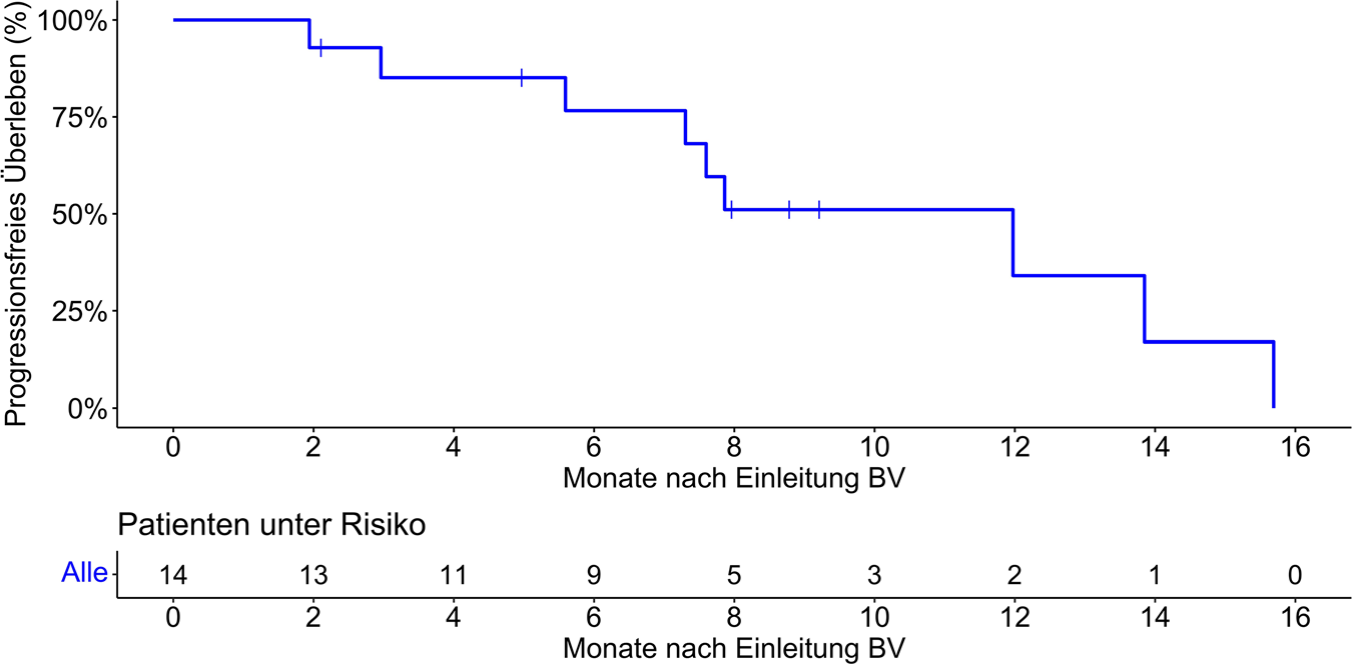
Kaplan‐Meier‐Diagramm des progressionsfreien Überlebens der Gesamtkohorte (n = 14) nach Therapie mit Brentuximab vedotin und simultaner oder sequenzieller Strahlentherapie. *Abk*.: BV, Brentuximab vedotin.

**ABBILDUNG 2:**
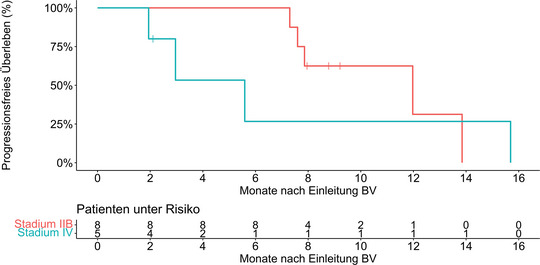
Kaplan‐Meier‐Diagramm des progressionsfreien Überlebens der Subgruppen mit Erkrankungsstadium IIB und IV (n = 13) nach Therapie mit Brentuximab vedotin und simultaner oder sequenzieller Strahlentherapie. *Abk*.: BV, Brentuximab vedotin

**ABBILDUNG 3:**
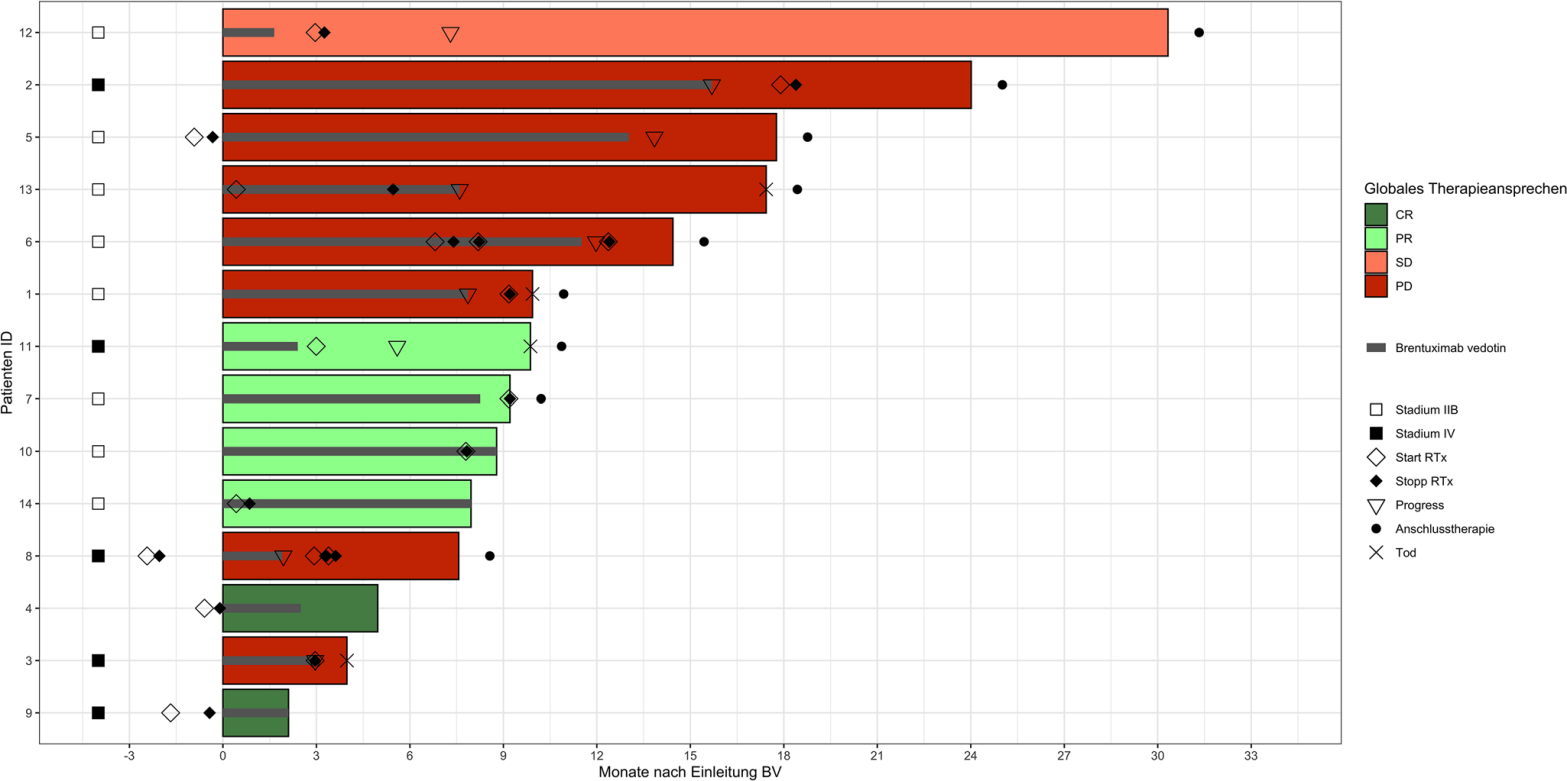
Swimmer‐Plot, der den therapeutischen Verlauf seit Beginn der Behandlung mit Brentuximab vedotin in der Gesamtkohorte (n = 14) zeigt. *Abk*.: BV, Brentuximab vedotin; CR, komplette Remission; ID, Identifikation; PD, progrediente Erkrankung; PR, partielle Remission; RTx, Strahlentherapie; SD, stabile Erkrankung

Nach einer medianen Nachbeobachtungszeit von 14,4 Monaten (Interquartilsbereich [IQR] 8,0–24,0 Monate) betrug das mediane PFS 12,0 Monate (95%‐KI 7,3 bis nicht verfügbar [NA]) mit einer 1‐Jahres‐PFS‐Rate von 34,0% (95%‐KI 12,9–90,1) (Abbildung 1). Eine Untergruppenanalyse der Krankheitsstadien IIB und IV ergab ein medianes PFS von 12,0 Monaten (95%‐KI 7,9–NA) für Stadium IIB und 5,6 Monaten (95%‐KI 3,0–NA) für Stadium IV. Die 1‐Jahres‐PFS‐Raten lagen bei 31,3% (95%‐KI 7,1–100) beziehungsweise 26,7% (95%‐KI 5,1–100) (Abbildung 2). Vier Patienten verstarben während der Nachbeobachtungszeit, davon ein Lymphom‐assoziierter Tod. Während der Nachbeobachtung erhielten neun Patienten (64%) mindestens eine Anschlusstherapie, darunter Mogamulizumab, Gemcitabin, MTX, PUVA, Bexaroten, TSEBT, Resminostat oder pegyliertes liposomales Doxorubicin. Tabelle 2 fasst die Therapieergebnisse zusammen, Abbildung 3 gibt einen Überblick über die jeweiligen individuellen Therapieverläufe.

### Unerwünschte Ereignisse

In unserer Kohorte hatten 71% (10/14) der Patienten ein AE beliebigen Schweregrades, wobei bei sechs Patienten mehr als eine AE dokumentiert wurde. Schwerwiegende AE (≥ Grad 3) traten bei drei Patienten auf: Neutropenie, Lymphopenie und Radiodermatitis (Tabelle 3). Der Patient mit Radiodermatitis (ID 2) musste die RTx nach 11 Fraktionen bei einer kumulativen Dosis von 22 Gy vorzeitig abbrechen; die Bestrahlung war 67 Tage nach der letzten Gabe von BV begonnen worden. Die beiden Patienten mit hochgradigen hämatologischen Toxizitäten hatten jeweils eine hochdosierte RTx (30 Gy) erhalten. Einer von ihnen (ID 4) entwickelte zunächst eine leichte Radiodermatitis (Grad 1); seine RTx (30 Gy) war drei Tage vor Einleitung von BV abgeschlossen worden. Aufgrund der hohen Tumorlast kam es bei diesem Patienten kurz nach der ersten Gabe von BV zu einem Tumorlyse‐Syndrom mit akutem Nierenversagen Grad 1 sowie zu einer Hämatotoxizität Grad 3 mit Leukopenie und febriler Neutropenie. Betrachtet man das gewählte Kombinationsregime, so traten bei zwei von vier Patienten, die ihre RTx drei beziehungsweise zehn Tage vor Einleitung von BV beendet hatten, AE auf. Bei Patienten, die eine RTx nach Beendigung von BV erhielten, wurden für 57% (4/7) AE jeden Schweregrades dokumentiert. In der Kohorte der Patienten, die eine simultane Gabe von RTx und BV erhielten, traten bei 80% (4/5) AE jeden Schweregrades auf.

**TABELLE 3:** Unerwünschte Ereignisse.

	Patienten n/n (%)
***AE während und 4 Wochen nach der Kombinationstherapie***	
Jeden Schweregrades	10/14 (71)
≥ Grad 3	3/14 (21)
≥ zwei AE (jeden Schweregrades)	6/14 (43)
***Betroffene Organsysteme***	
Haut	5/14 (36)
Leber/Bauchspeicheldrüse	3/14 (21)
Niere	2/14 (14)
Nervensystem	6/14 (43)
Hämatologisch	3/14 (21)
Andere^*^	4/14 (29)

Prozentwerte ergeben möglicherweise nicht genau 100%, da gerundet wurde.

* Andere dokumentierte unerwünschte Ereignisse beinhalteten Müdigkeit, Fieber, Gewichtsverlust, Hyperurikämie und eine SARS‐CoV‐2‐Infektion.

*Abk*.: AE, unerwünschtes Ereignis

Bei den dokumentierten AE handelte es sich um für BV und/oder RTx bekannte AE. Hinweise auf ungewöhnliche/unerwartete Toxizitäten oder Verschlimmerungen von AE aufgrund der Kombinationstherapie lagen nicht vor. Neurologische AE traten bei 43% (6/14) der Patienten auf und bestanden aus einer peripheren Neuropathie (PNP). Zu den hautbedingten AE gehörten Exantheme, Radiodermatitis, Weichteilinfekte und Alopezie. Zu den hämatologischen AE gehörten hauptsächlich Neutro‐, Leuko‐ und/oder Lymphopenie, während zu den hepatischen/pankreatischen AE erhöhte Leber‐ oder Pankreasenzyme zählten (Tabelle 3). Bei allen Patienten mit PNP war eine Änderung der Dosierung und/oder der Intervalle von BV aufgrund der Nebenwirkungen erforderlich. In unserer Kohorte kam es zu keinem vorzeitigen Absetzen von BV.

## DISKUSSION

Die therapeutischen Optionen für CD30‐positives CTCL sind nahezu so vielfältig wie die Erkrankung selbst. Bisher liegen lediglich kleine Fallserien zur gleichzeitigen oder sequenziellen Kombination von BV und RTx vor.^16^ Wir präsentieren hier eine retrospektive Analyse von 14 Patienten mit CD30‐positivem CTCL aus sechs deutschen Tumorzentren, die eine Kombinationstherapie mit BV und RTx erhielten. Die Dosierung und der Zeitpunkt der jeweiligen Therapien variierten zwischen den Zentren und den einzelnen Patienten, je nach Entscheidung des Arztes. Während viele randomisierte Studien ausgewählte Patienten mit Einschränkungen in Bezug auf vorherige Therapien oder das Krankheitsstadium einschließen, bestand unsere Patientenkohorte hauptsächlich aus MF‐Patienten im Krankheitsstadium IIB und IV und einem Patienten mit PCTL‐Tfh.

Hinsichtlich der Verträglichkeit des kombinierten Regimes traten in unserer Kohorte keine ungewöhnlichen oder verstärkten AE auf. Während des Behandlungszeitraums hatten 71% (10/14) aller Patienten ein AE beliebigen Schweregrades. Hinsichtlich des Behandlungssettings erlitt etwa die Hälfte der Patienten mit sequenzieller RTx AE, im Gegensatz zu 80% (4/5) bei simultaner RTx. Inwieweit die zeitliche Nähe der beiden Therapien zu einem vermehrten Auftreten von Nebenwirkungen führt, lässt sich anhand der erhobenen Daten nicht feststellen. Darüber hinaus ist unklar, inwieweit BV vor RTx zu einer Verschlimmerung von RTx‐assoziierten AE führt. Eine mögliche Radiosensibilisierung von MMAE wurde bereits beschrieben, jedoch ist die Dauer dieses Effekts noch unklar.^17^ Eine PNP niedrigen Grades (Grad 1–2), ein sehr häufiges unerwünschtes Ereignis unter BV, trat bei 43% (6/14) der Patienten auf. Bei allen Patienten mit PNP war eine Anpassung der Dosis und/oder des Intervalls von BV erforderlich, jedoch musste die Behandlung nicht dauerhaft abgebrochen werden. Im Vergleich dazu hatten 67% der Patienten im BV‐Studienarm der ALCANZA‐Studie eine PNP, von denen 52% mindestens eine BV‐Dosisanpassung benötigten.^4^ Eine für RTx häufige AE in unserer Kohorte war die Radiodermatitis, die bei einem Patienten (ID 2) zu einem vorzeitigen Abbruch der RTx, nach bereits erfolgter Applikation einer Gesamtstrahlendosis von 22 Gy (11 x 2 Gy), führte. Die Zeitspanne zwischen BV und RTx betrug in diesem Fall 67 Tage. Weitere hautbezogene AE wie Alopezie, Weichteilinfektionen und Exantheme ließen sich beiden Behandlungen zuordnen; Angaben zu Zeitpunkt oder Lokalisation des Auftretens lagen jedoch nicht vor. Zu den hämatologischen AE zählten Leuko‐, Lympho‐ und Neutropenien. Keiner der Patienten mit hämatologischen Nebenwirkungen erhielt eine TSEBT. Hämatologische AE sind bei BV häufig, insbesondere bei systemischen hämatolymphoiden Neoplasien. Ein zusätzlicher negativer Effekt der RTx kann jedoch nicht sicher ausgeschlossen werden, wie ein Patientenfall (ID 4) zeigt, bei dem die Therapie mit BV drei Tage nach Abschluss einer RTx (30 Gy) begonnen wurde. Kurz danach kam es zu einem Tumorlysesyndrom. Hier könnte die Bestrahlung der Tumormasse in Kombination mit BV die erwartete Tumorlyse durch die BV‐Monotherapie verschlimmert haben. Ein weiterer Patient entwickelte unter gleichzeitiger Therapie mit BV und hochdosierter RTx (30 Gy) schwere hämatologische Toxizitäten (Grad 3). Kürzlich berichteten Wu et al. über eine Fallserie von 44 Lymphompatienten, die gleichzeitig mit BV und RTx behandelt wurden. Darunter befanden sich 22 Patienten mit MF und vier mit einem anderen CTCL‐Subtyp. Schwere hämatologische Nebenwirkungen (≥ Grad 2) traten bei 20% der Gesamtkohorte auf. Ferner konnte ein Zusammenhang zwischen den aufgetretenen hämatologischen AE und der verwendeten Strahlendosis festgestellt werden.^18^

Ein globales Ansprechen einer CR oder PR erreichten 43% (6/14) der Patienten unserer Kohorte. Im Vergleich dazu lag die mindestens 4 Monate anhaltende ORR (ORR4) in der ALCANZA‐Studie bei 65,6% (42/64) für BV allein. Das mediane PFS in unserer Kohorte betrug 12,0 Monate (95%‐KI 7,3–NA), verglichen mit 16,7 Monaten (95%‐KI 15,4–21,6) in der ALCANZA‐Studie.^4^ Ein fortgeschrittenes Tumorstadium und ein langjähriger Krankheitsverlauf in Verbindung mit verschiedenen vorherigen systemischen Therapien sowie eine kleine Patientenkohorte könnten mögliche Erklärungen für diese Diskrepanz sein. Da BV als Zweitlinientherapie zugelassen ist, erhielten alle Patienten in unserer Kohorte mindestens eine vorherige systemische Therapie, wobei 86% (12/14) Bexaroten und 43% (6/14) MTX erhielten. In der ALCANZA‐Studie wurden Patienten mit BV nach MTX oder Bexaroten ausgeschlossen.^4^ Ein Patient in unserer Kohorte (ID 8) erhielt eine vorherige Behandlung mit BV und erreichte hierbei eine CR. Einige Monate nach dem Absetzen der Behandlung kam es jedoch zu einem Krankheitsprogress, was zu einer erneuten Behandlung mit BV in Kombination mit sequenzieller RTx führte. Bei dieser erneuten Behandlung erreichte der Patient nur eine PD als globales Ansprechen. Die Informationen über eine erneute Behandlung mit BV sind noch sehr begrenzt. Muniesa et al. berichteten über eine Fallserie von 13 Patienten, die eine erneute Behandlung mit BV erhielten und bei denen eine ORR von 54% erreicht wurde, wobei 23% eine CR erzielten.^19^

In unserer Kohorte erreichten 93% (13/14) eine CR oder PR als BOR während BV. Hiervon hatten nur 43% (6/14) ein anhaltendes Ansprechen (CR oder PR) bis zur Beendigung von BV. Neun Patienten der Gesamtkohorte erhielten während der Nachbeobachtung mindestens eine Anschlusstherapie. Ein anfängliches Ansprechen bis hin zu einer klinischen CR nach RTx wurde auch in einem Fallbericht von Floyd et al. beschrieben.^14^ In diesem Fall erhielt ein Patient mit primär kutanem anaplastischem großzelligem Lymphom eine Kombinationstherapie aus BV und RTx. Nach einer anfänglichen CR kam es etwa 7 Monate später zu einem Wiederauftreten der Erkrankung. Eine weitere Fallserie mit sechs CTCL‐Patienten zeigte eine Krankheitsstabilisierung unter BV in Kombination mit hautgerichteten Therapien wie lokaler Bestrahlung, Tumorexzision oder PUVA.^20^ In unserer Kohorte wurde eine RTx meist lokalisiert eingesetzt, um neu entstandene Tumoren zu stabilisieren. Drei Patienten unserer Gesamtkohorte erhielten eine TSEBT. Aufgrund unseres kleinen Patientenkollektivs und des heterogenen Real‐World‐Settings können keine validierten Informationen über den Zusammenhang zwischen dem globalen Ansprechen und der verwendeten RTx‐Modalität/Dosierung (niedrige vs. hohe Dosis) gegeben werden. Innerhalb der EORTC‐Gruppe für kutane Lymphome laufen derzeit gemeinsame Studien zur Evaluation von Bestrahlungsprotokollen unter Berücksichtigung der Heterogenität der derzeitigen Bestrahlungspraxis in den jeweiligen Zentren.^21^ Die Entwicklung evidenzbasierter Empfehlungen für Dosierung, Fraktionierung und Technik sowie für geeignete Kombinationstherapien ist für eine Optimierung und Standardisierung der Behandlung von kutanen Lymphomen erforderlich.^8^

Zusammenfassend zeigen unsere Daten, dass die Behandlung fortgeschrittener CD30‐positiver CTCL mit BV in sequenzieller oder simultaner Kombination mit RTx (entweder TSEBT oder lokalisierte RTx) eine gut durchführbare und verträgliche therapeutische Option ist, die keine Anzeichen für verstärkte/unerwartete Toxizitäten aufweist. Im Hinblick auf die Wirksamkeit kann eine additive RTx zu einer Stabilisierung der Erkrankung und zur Behandlung neu entstandener Läsionen unter BV beitragen. Unsere retrospektive Analyse wies durch die unsystematische Dokumentation, die heterogenen Behandlungsschemata in einem Real‐World‐Setting, die geringe Patientenzahl und die bevorzugte Einbeziehung von Patienten im fortgeschrittenen Stadium erhebliche Einschränkungen auf. Dennoch sind das vorgeschlagene Konzept und die hier vorgestellten vorläufigen Daten vielversprechend für künftige prospektive Untersuchungen. Das Hauptziel wird sein, die Wirksamkeit und Verträglichkeit des kombinierten Behandlungsansatzes sowie den optimalen Zeitpunkt und die Dosierung von BV und RTx systematisch zu evaluieren.

## FINANZIERUNG

Patrick Schummer und Caroline Glatzel werden durch TWINSIGHT, ein Clinician‐Scientist‐Programm der Medizinischen Fakultät der Universität Würzburg, gefördert, das von der Else Kröner‐Fresenius‐Stiftung finanziert wird.

## DANKSAGUNG

Open access Veröffentlichung ermöglicht und organisiert durch Projekt DEAL.

## INTERESSENKONFLIKT

G.D. erhielt Honorare und Reisekostenzuschüsse von Takeda, Helsinn, Recordati Rare Diseases und Kyowa Kirin außerhalb der eingereichten Arbeit. U.W. war als Berater tätig und/oder erhielt Honorare und/oder Reisekostenzuschüsse von Takeda, Helsinn, Recordati Rare Diseases, Stemline Therapeutics und Kyowa Kirin, jeweils außerhalb der eingereichten Arbeit. J.P.N. erhielt Reise‐ und Kongressteilnahmekosten von TEVA und Novartis sowie Beratungshonorare von TEVA, Almirall, Biogen, Novartis, Kyowa Kirin, Innate Pharma, Takeda, Actelion, UCB Pharma und Recordati außerhalb der eingereichten Arbeit. M.G. war als Berater tätig und/oder erhielt Vortragshonorare und/oder Reisekostenzuschüsse von Almirall, Argenx, Boehringer Ingelheim, Biotest, GSK, Janssen, Leo Pharma, Lilly, Novartis und UCB Pharma, jeweils außerhalb der eingereichten Arbeit. M.W. erhielt Honorare und Reisekostenzuschüsse von Takeda, Recordati Rare Diseases, Stemline Therapeutics und Kyowa Kirin außerhalb der eingereichten Arbeit. P.S., C.G., Ph.S., I.L., S.H. und R.S. erklären, dass kein Interessenkonflikt besteht.
